# 

**Published:** 1865-06

**Authors:** 


Volume XXII. Number 6.

### THE CHICAGO

### MEDICAL JOURNAL

De LASKIE MILLER, M. D.,

EPHRAIM INGALS, M. D.,

Editors and Proprietors.

JUNE, 1865.

CHICAGO:
J. C. W. BAILEY, BOOK AND JOB PRINTER, 130 CLARK STREET.
1865.

